# Mutation of N-linked glycosylation at Asn548 in CD133 decreases its ability to promote hepatoma cell growth

**DOI:** 10.18632/oncotarget.4115

**Published:** 2015-05-12

**Authors:** Ying Liu, Shifang Ren, Liqi Xie, Chunhong Cui, Yang Xing, Chanjuan Liu, Benjin Cao, Fan Yang, Yinan Li, Xiaoning Chen, Yuanyan Wei, Haojie Lu, Jianhai Jiang

**Affiliations:** ^1^ Key Laboratory of Glycoconjuates Research, Ministry of Public Health and Gene Research Center, Department of Biochemistry and Molecular Biology, Shanghai Medical College of Fudan University, Shanghai, People's Republic of China; ^2^ Institutes of Biomedical Sciences of Fudan University, Shanghai, People's Republic of China

**Keywords:** N-linked glycosylation, CD133, hepatoma, cell growth

## Abstract

The membrane glycoprotein CD133 is a popular marker for cancer stem cells and contributes to cancer initiation and invasion in a number of tumor types. CD133 promotes tumorigenesis partly through an interaction between its phosphorylated Y828 residue and the PI3K regulatory subunit p85, and the interaction with β-catenin. Although CD133 glycosylation is supposed to be associated with its function, the contribution of N-glycosylation to its functions remains unclear. Here we analyzed the exact site(s) of N-glycosylation in CD133 by mass spectrometry and found that all eight potential N-glycosylation sites of CD133 could be indeed occupied by N-glycans. Loss of individual N-glycosylation sites had no effect on the level of expression or membrane localization of CD133. However, mutation at glycosylation site Asn548 significantly decreased the ability of CD133 to promote hepatoma cell growth. Furthermore, mutation of Asn548 reduced the interaction between CD133 and β-catenin and inhibited the activation of β-catenin signaling by CD133 overexpression. Our results identified the characteristics and function of CD133 glycosylation sites. These data could potentially shed light on molecular regulation of CD133 by glycosylation and enhance our understanding of the utility of glycosylated CD133 as a target for cancer therapies.

## INTRODUCTION

CD133 (prominin-1) is a five-transmembrane domain glycoprotein located on apical plasma membrane protrusions [1-6]. It has been widely used as a cell surface marker to identify and isolate cancer stem cells from various tissues including the brain [7, 8], prostate [9, 10], liver [11, 12] and colon [13-15]. Increasing evidence has shown that CD133 contributes to cancer initiation and invasion, raising the possibility that CD133 is a molecular target for effective cancer therapies. For example, knockdown of CD133 decreases the colony-forming ability of human hepatocellular carcinoma cells [16]. CD133 promotes colon cancer cell proliferation through activating β-catenin signaling [17]. Recently, we showed that CD133 promotes self-renewal and tumorigenesis of glioma stem cells partly through an interaction between its phosphorylated Y828 residue and the PI3K regulatory subunit p85 [18]. Although the role of CD133 in cancer development is being explored gradually, the mechanism involved in its functions remains largely unknown.

N-linked glycosylation, one of the most common co/post-translational modifications, has important biological functions in proteins folding, trafficking, cell-cell interactions, and signal transduction [19, 20]. CD133 protein is predicted to contain eight N-linked glycosylation sites within its putative extracellular domains [21, 22]. Kemper has shown that CD133 mRNA and protein were not decreased when cancer stem cell differentiated, but the AC133 epitope is lost [23]. Thus, identification of CD133 glycosylation sites or structures may be a crucial step to define the potential role of CD133 in normal and cancer stem cells. Our previous data has shown that alpha 2,3-sialylation increases the stability of CD133 [24]. Recently, Mak has identified a glycosyltransferase MGAT4C for CD133 complex N-glycan processing in a shRNA screening. Moreover, loss of individual N-glycosylation sites had no effect on the stability or transmembrane location of CD133 [25]. However, the contribution of N-glycosylation to the functions of CD133 remains elusive.

In this study, we analyzed the exact site(s) of N-glycosylation in CD133 by mass spectrometry (MS) and found that CD133 contained nine N-linked glycosylation sites (Asn206, Asn220, Asn274, Asn395, Asn414, Asn548, Asn580, Asn729, and Asn730). Mutation experiments showed that lack of single N-glycosylation sites in CD133 did not change its expression or cell surface localization. Interestingly, loss of N-linked glycosylation at Asn548 led to a reduction in CD133-induced cell proliferation and decreased the ability of CD133 to associate with β-catenin and activate β-catenin signaling pathway.

## RESULTS

### Identification of N-glycosylation sites in CD133 by MS

**Figure 1:**
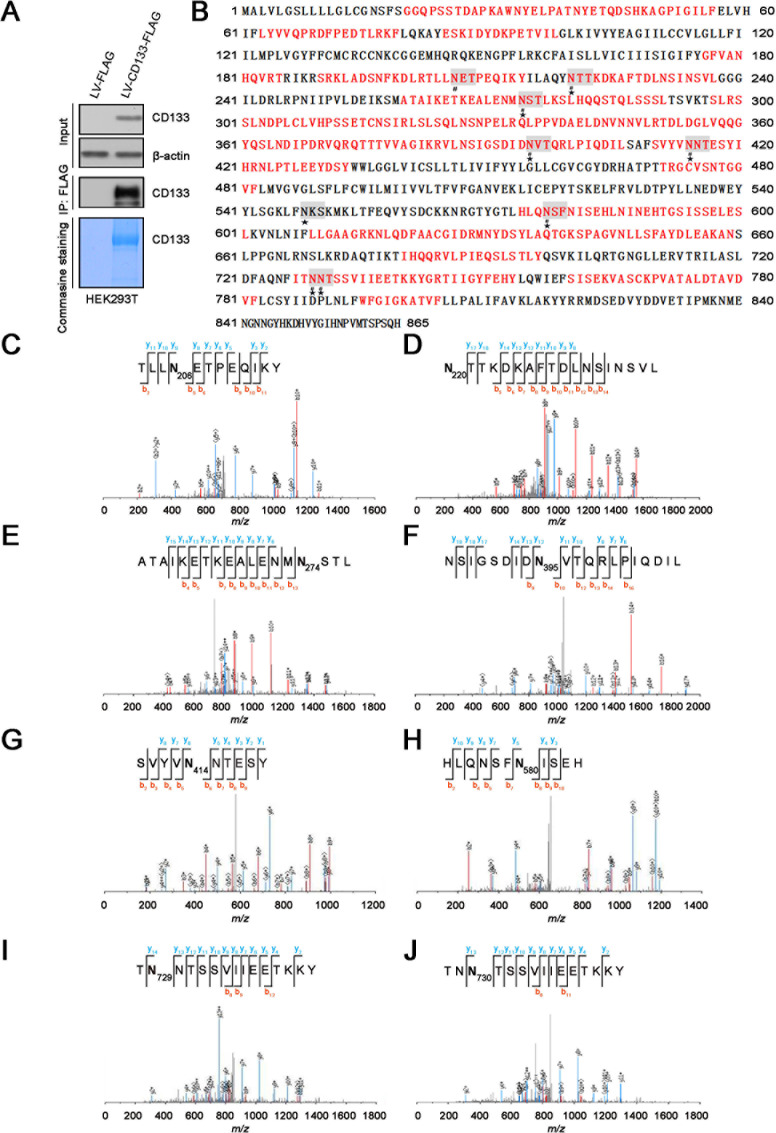
Site-specific characterization of N-glycosylation in CD133 by MS analysis **A.** CD133 protein immunoprecipitated from HEK293T cells expressing FLAG-tagged CD133 was stained with Coomassie blue R-250 or analyzed by SDS-PAGE followed by western blotting with an anti-CD133 antibody. **B.** Glycosylation sites and sequence coverage of CD133 identified by MS (related to Figure 1C–J). The N-glycosylation sites and peptides of CD133 were identified by chymotrypsin (red). The N-glycosylation sites with the amino acid number are gray. ★ indicates potential N-glycosylation consensus sites (Asn-X-Ser/Thr), # represents identification of N-glycosylation site occupancy by LC-MS/MS. **C.** MS spectrum of the 0.98 Da increase glycopeptide (R.TLLNETPEQIKY.I, [(M+2H)^2+^ at *m/z* 117.02]). **D.** Y.NTTKDKAFTDLNSINSVL.G, [(M+2H)^2+^ at *m/z* 117.02]. **E.** M.ATAIKETKEALENMNSTL. K, [(M+2H)^2+^ at *m/z* 117.02]. **F.** L.NSIGSDIDNVTQRLPIQDIL.S, [(M+2H)^2+^ at *m/z* 117.02]. **G.** F.SVYVNNTESY.I, [(M+2H)^2+^ at *m/z* 117.02]. **H.** L.HLQNSFNISEH.L, [(M+2H)^2+^ at *m/z* 117.02]. **I.** I.TNNTSSVIIEETKKY.G, [(M+2H)^2 +^ at *m/z* 117.02], and **J.** I.TNNTSSVIIEETKKY.G, [(M+2H)^2 +^ at *m/z* 117.02] from the PNGase F-treated sample.

To obtain abundant CD133 proteins for identification of N-glycosylation sites by MS, an expression vector encoding human CD133 fused with a C-terminal FLAG tag was transiently transfected into HEK293T cells. We chose HEK293T cells because they allow proper glycosylation of CD133 and cell surface AC133 expression [25]. Next, CD133 glycoprotein was purified by immunoprecipitation (IP) from the HEK293T cells expressing CD133-FLAG and stained with Coomassie blue R-250 (Figure 1A). Purified CD133 protein was reduced and alkylated in solution and then digested by chymotrypsin. N-glycosylation sites of CD133 were identified by MS after treatment with peptide-N-glycosidase F (PNGase F). MS analysis identified eight N-glycosylation sites: Asn206, Asn220, Asn274, Asn395, Asn414, Asn580, Asn729, and Asn730 (Figure 1C–1J). However, this approach did not yield full sequence coverage or guaranteed identification of all N-glycosylation sites (Figure 1B). Thus, N-glycosylation sites of CD133 were further identified by matrix-assisted laser desorption/ionization (MALDI) MS analysis as described previously [26, 27].

**Figure 2:**
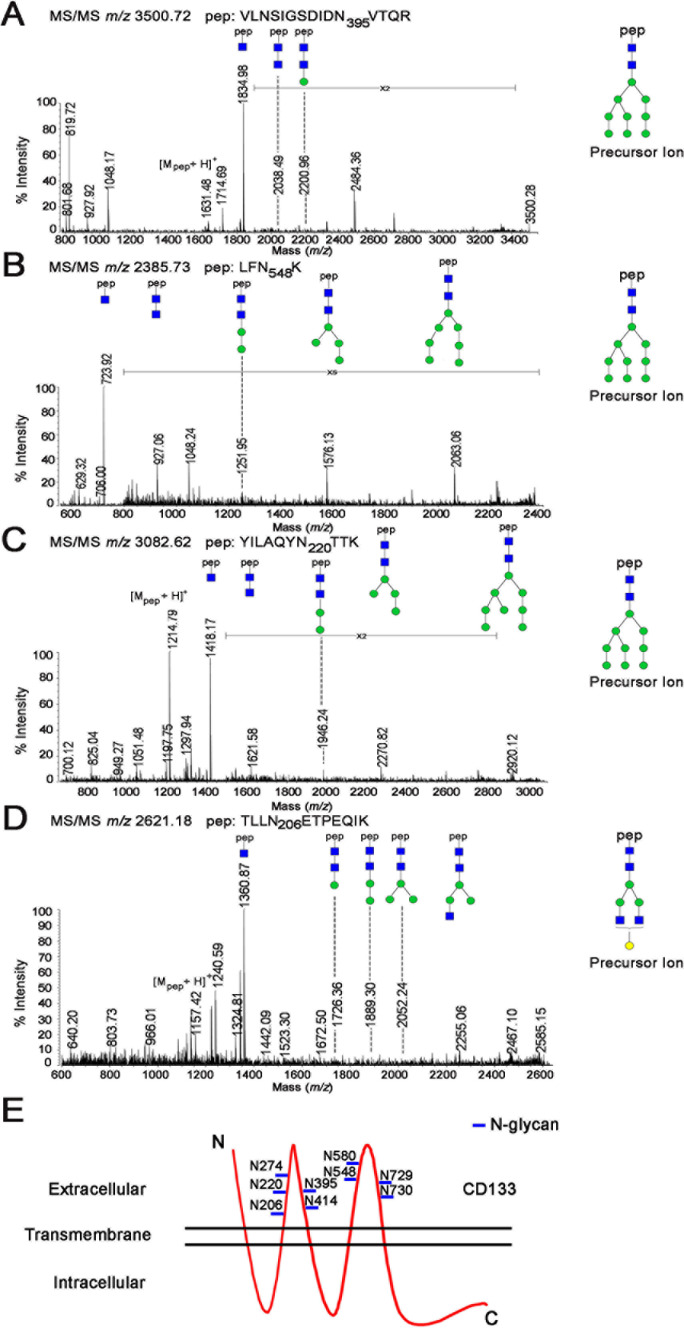
MS/MS spectrum of the identified glycopeptide from CD133 **A.**–**D.** MS/MS spectra of the glycopeptides identified at Asn395 **A.**, Asn548 **B.**, Asn220 **C.**, and Asn206 **D.** contained (HexNAc)2Hex9 and (HexNAc)4Hex4. Symbols used are as follows: blue box, N-acetylhexosamine (HexNAc); green circle, mannose (Hexose); yellow circle, galactose (Hexose); pep, peptide. **E.** Model of CD133 N-glycosylation. CD133 protein has an extracellular N-terminus, cytoplasmic C-terminus, and two very large extracellular loops containing nine N-glycosylation sites (marked with a blue line).

As shown in Figure 2, the most intense signal at *m/z* 1834.98 as a [Mpep+203+H]^+^ fragment, together with the signal at *m/z* 1631.48 revealed cleavage between the Asn and first N-acetyl-D-glucosamine (GlcNAc) in the core glycan structure. Moreover, the ^0,2^X-ring cleavage of the innermost N-acetylglucosamine generated a [Mpep+83+H]^+^ ion at *m/z* 1714.69, which further confirmed that the mass of the peptide moiety was 1631.48 Da. Accordingly, the corresponding peptide sequence was assigned as VLNSIGSDIDN395VTQR with a theoretical mass of 1631.77 Da. Therefore, we identified a glycosylation site at Asn395 in CD133 (Figure 2A). Using a similar analytical method, we further characterized the N-glycosylation sites of Asn548 (Figure 2B), Asn220 (Figure 2C), and Asn206 (Figure 2D). Collectively, CD133 contained nine N-linked glycosylation sites (Asn206, Asn220, Asn274, Asn395, Asn414, Asn548, Asn580, Asn729, and Asn730) (Figure 2E).

### Expression and cellular localization of CD133 or its N-glycosylation mutants

**Figure 3:**
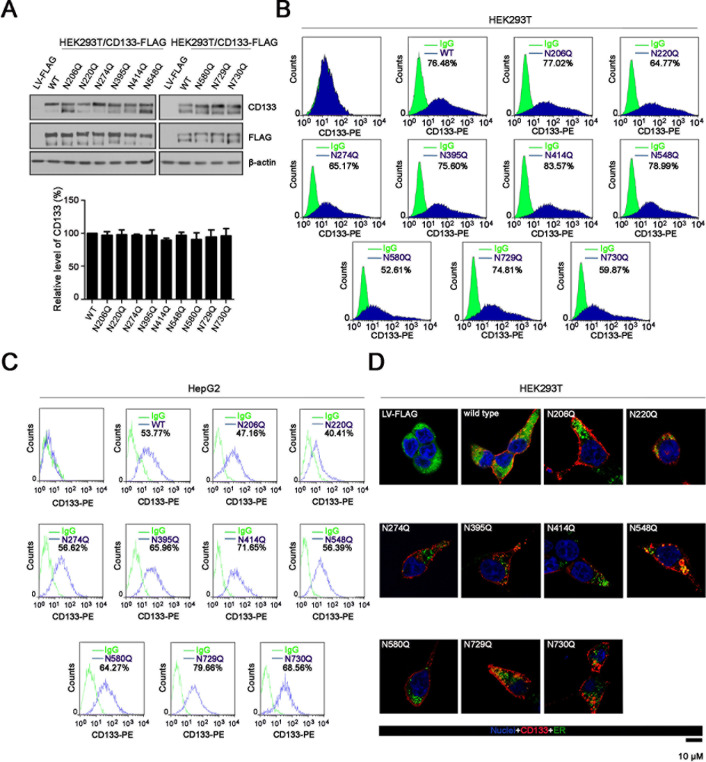
Expression and cellular localization of wild-type CD133 or its N-glycosylation site mutant **A.** Lysates of HEK293T cells expressing wild-type CD133 or the single-site N-glycosylation CD133 mutant were analyzed by SDS-PAGE followed by western blotting with an anti-CD133 antibody. β-actin was used as a loading control. The experiment was performed as a biological triplicate, and a representative replicate is shown (upper panel). Expression levels of the single-site N-glycosylation CD133 mutant were normalized to β-actin and are relative to wild-type CD133 (lower panel). Results are expressed as mean ± SD from three separate experiments. **B.** and **C.** HEK293T **B.** or HepG2 **C.** cells expressing either wild-type or CD133 mutant were stained with a PE-labeled anti-AC133 antibody followed by flow cytometry (*n* = 3). Green line, control IgG staining; blue line, CD133 staining. CD133-positive cell rates are shown. Representative flow cytometry datas from three independent experiments are shown. **D.** Immunofluorescence staining to determine the localization of CD133 (red) in HEK293T cells expressing wild-type CD133 or its N-glycosylation site mutant. Cells were co-stained with an ER-specific dye (green). Nuclei were stained with Hoechst 33258. Scale bar: 10 μM.

To investigate the significance of N-glycosylation in CD133 function, we generated single-site glycosylation mutants by substituting asparagine (N) with glutamine (Q) in the nine N-glycosylation sites. Western blots showed that all nine N-glycosylation sites mutants were expressed in HEK293T cells, at similar levels to wild-type CD133 (Figure 3A). Moreover, mutation of individual N-glycosylation sites had no effect on cell surface expression of CD133 in HEK293T or HepG2 cells (Figure 3B and 3C). Consistent with the above findings, immunofluorescence staining assay showed that both wild-type CD133 and individual N-glycosylation mutants were primarily localized to the plasma membrane (Figure 3D).

### Mutation of CD133 at Asn548 reduces its ability to promote hepatoma cell growth

**Figure 4:**
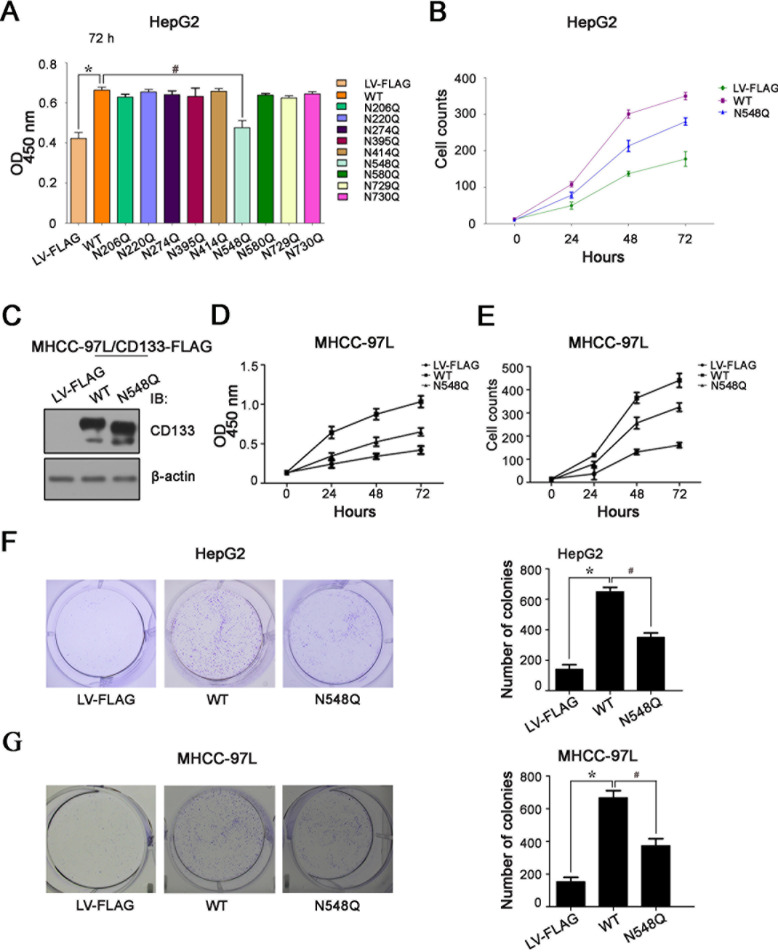
Mutation of CD133 at Asn548 reduces its ability to promote hepatoma cell growth **A.** and **B.** Cell proliferation rate of HepG2 cells infected lentivirus expressing wild-type CD133 or single-site N-glycosylation mutant was determined by MTT assay at 72h **A.** and cell counting **B.** in different time points. Results are expressed as mean ± SD from three separate experiments. *P < 0.05 *vs* LV-FLAG, ^#^*P* < 0.05 *vs* wild-type CD133. **C.–E**. Western blot analysis of CD133 expression in MHCC-97L cells infected with lentivirus expressing wild-type CD133 or N548Q mutant. β-actin served as a loading control **C.**. Mutation of Asn548 impaired CD133-induced MHCC-97L cell growth as indicated by MTT assay **D.** and cell counting **E.**. Data represent the means ± SD derived from three independent experiments. **F.** and **G.** Colony formation was detected using HepG2 cells **F.** and MHCC-97L **G.** cells expressing wild-type CD133 or the N548Q mutant. Representative images are shown in the left panel **F**. and **G**., and the number of colonies expressed as the mean ± SD of three separate experiments is shown in the right panel **F.** and **G.**. *P < 0.05 *vs* LV-FLAG, ^#^*P* < 0.05 *vs* wild-type CD133.

We next determined the effects of single N-glycosylation site mutations on the growth of hepatoma cells by 3-[4,5-dimethylthiazol-2-yl]-2,5-diphenyltetrazolium bromide (MTT) assay and cell counting. Consistent with previous findings that CD133 increases hepatoma cell growth [28, 29], the proliferation rate was 2-3-fold higher in HepG2 cells overexpressing CD133 compared with control cells (Figure 4A and 4B). In contrast, HepG2 cells overexpressing the N584Q mutant exhibited a striking decrease in cell proliferation compared with the cells overexpressing CD133 (Figure 4A). This finding was also confirmed in MHCC-97L hepatoma cells (Figure 4C–4E).

To verify the effect of N548Q mutation on hepatoma cell growth, clonogenic assays were performed. The results showed that hepatoma cells overexpressing the N548Q mutant displayed much fewer and smaller colonies compared with cells overexpressing CD133 (Figure 4F and 4G).

### Mutation at glycosylation site Asn548 decreases the binding of CD133 to β-catenin

**Figure 5:**
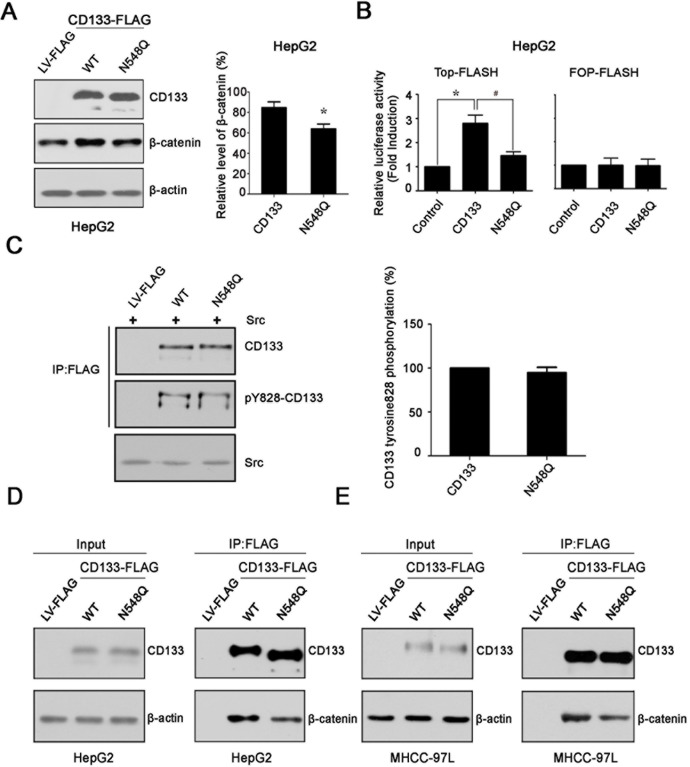
Mutation at glycosylation site Asn548 decreases the binding of CD133 to β-catenin **A.** Western blotting was used to determine the effect of the N548Q mutation on CD133-induced β-catenin expression (left panel) and three independent experiments (mean ± SD) (right panel) are shown. *P < 0.05 *vs* wild-type CD133. **B.** Control or wild-type CD133 or FLAG-tagged N548Q were transiently co-transfected into HepG2 cells with TOP-FLASH or FOP-FLASH. Luciferase activity was determined as described in supplementary information. *P < 0.05 *vs* LV-FLAG, ^#^*P* < 0.05 *vs* wild-type CD133. **C.** HEK293T cells were transfected with either an empty vector, wild-type CD133, or the N548Q mutant together with Src. At 48h post-transfection, cells lysates were immunoprecipitated (IP) with an anti-FLAG antibody (Ab), the immunoprecipitates were immunoblotted with antiphosphotyrosine antibody (PY828), and the same membrane was reprobed with anti-CD133 antibody. Levels of transfected Src were monitored in lysates by immunoblotting using an anti-Src monoclonal antibody (left panel). Quantitative results are illustrated for left panel (right panel). **D.** and **E.** Immunoprecipitation (IP) analysis to determine the effect of the N548Q mutation on the interaction between CD133 and β-catenin in hepatoma cells. Lysates of HepG2 **D.** cells and MHCC-97L **E.** cells expressing FLAG tagged wild-type CD133 or the FLAG-tagged N548Q mutant were subjected to IP using an anti-FLAG antibody, followed by immunoblotting (IB) with anti-CD133 or anti-β-catenin antibodies.

It has been shown that CD133 promotes cancer cell proliferation through an interaction with β-catenin and an increase in β-catenin stability [17]. To examine the mechanism by which N548Q mutation reduced the ability of CD133 to promote hepatoma cell growth, we first determined the effect of N548Q mutation on the level of β-catenin protein. The results showed that overexpression of CD133 in HepG2 cells led to an increase in endogenous β-catenin levels. The increased levels of β-catenin protein were significantly decreased in cells overexpressing the N548Q mutant compared with cells overexpressing CD133 (Figure 5A). TOP/FOP luciferase reporter assay has been widely used to measure β-catenin signaling activity [30, 31]. We next examined the effect of N548Q mutation on activation of β-catenin signaling using the TOP/FOP luciferase reporter assay. The results indicated that CD133 remarkably increased the TOP-FLASH activity in HepG2 cells. By contrast, mutation of Asn548 resulted in a significant reduction of CD133-induced TOP-FLASH activation (Figure 5B). A similar effect of N548Q mutation on β-catenin expression and signaling was also observed in MHCC-97L cells (data not shown). Lastly, we analyzed the effect of N548Q mutation on the interaction between CD133 and β-catenin, and found that loss of N-glycosylation at Asn548 decreased the binding of CD133 to β-catenin in HepG2 and MHCC-97L cells (Figure 5D and 5E). Considering that CD133 promotes tumorigenesis partly through an interaction between its phosphorylated Y828 residue and the PI3K regulatory subunit p85 [18], we assessed the effect of N548Q mutation on CD133 Y828 phosphorylation. The results showed that Y828 phosphorylation level was similar between the cells overexpressing CD133 and the N548Q mutant (Figure 5C).

## DISCUSSION

The glycosylation status of CD133 is supposed to be correlated with cell differentiation and recognition of CD133 by the anti-AC133 antibody [32]. However, the specific glycosylation status of CD133 remains unclear. In this study, we performed characterization of the glycosylation sites in CD133 and showed that all eight potential N-glycosylation sites of CD133 could be indeed occupied by N-glycans.

N-linked glycosylation is critical for membrane proteins folding, co-assembly and stabilization [33-38]. In many instances, specific protein functions can be attributed to glycosylation at specific sites. For example, von Willebrand Factor (VWF) has 16 N-linked glycosylation sites and 4 mutations (N99Q, N857Q, N2400Q, and N2790Q) reduces VWF secretion [39]. Another example is Kaposi sarcoma-associated herpes virus-encoded interleukin-6 (vIL-6) that has two potential N-glycosylation sites (Asn78 and Asn89). Mutation of Asn89 disrupts the conformation of vIL-6 protein and diminishes the binding of vIL-6 to gp130 [40]. These findings suggest that N-glycosylation is necessary for maturation and the functional fate of membrane proteins. In this study, we found that lack of single N-glycosylation sites had no obvious effect on the expression level or membrane localization of CD133, which is similar to previous observations [25].

Our data showed that CD133 overexpression promoted hepatoma cell proliferation and significantly increased the number of colonies, suggesting that CD133 stimulates the growth of hepatoma cells. Importantly, mutation of Asn548 caused a marked reduction in cell viability and colony formation ability of HepG2 and MHCC-97L cells. Thus, N-linked glycosylation at Asn548 of CD133 is required for its ability to promote hepatoma cell proliferation. To the best of our knowledge, this is the first report demonstrating the functional role of N-glycosylation in CD133-induced hepatoma cell growth.

We have previously demonstrated that CD133 promotes tumorigenesis partly through the binding of its phosphorylated Y828 residue to the PI3K regulatory subunit p85 [18]. Our results showed that mutation of Asn548 did not change CD133 Y828 phosphorylation. Thus, we can exclude the possibility of a functional role of Asn548 glycosylation correlated with binding of phosphorylated Y828 residue to the PI3K regulatory subunit p85.

CD133 has been reported to promote cancer cell proliferation through the β-catenin signaling pathway. CD133 recruits HDAC6 to deacetylate β-catenin, resulting in its phosphorylation, stabilization, and nuclear localization to activate β-catenin signaling [17]. Our data revealed that mutation of Asn548 reduced CD133-induced β-catenin protein level and β-catenin signaling. Interestingly, mutation of Asn548 also significantly reduced the binding of CD133 to β-catenin. Growing documents have demonstrated that changes in glycosylation of surface receptors alter protein conformation which leads to impairing the binding of receptor to ligand or intracellular molecules, and ultimately disturbing signal transduction [40-44]. Thus, we speculate that CD133 without glycosylation at Asn548 might induce structural alternation that diminishes the binding of CD133 to β-catenin. We have to point out that we did not analyze the association of HDAC6 with N548Q mutant or applied a HDAC6 inhibitor to show whether loss of glycosylation at Asn548 increases β-catenin acetylation and phosphorylation. Notwithstanding this limitation, this study does suggest that loss of glycosylation at Asn548 decreased the ability of CD133 to activate β-catenin signaling pathway, ultimately led to a reduction in CD133-induced cell proliferation.

In conclusion, the present study identified the characteristics of CD133 glycosylation sites and showed that individual N-glycosylaton site was not essential for proper cell surface expression. Loss of N-glycosylation at Asn548 decreased CD133-induced hepatoma cell growth, reduced the association of CD133 with β-catenin, and inhibited the β-catenin signaling. Our findings potentially shed light on molecular regulation of CD133 by glycosylation and may enhance understanding of the utility of glycosylated CD133 as a molecular target for effective cancer therapies.

## MATERIALS AND METHODS

Detailed methods are described in supplementary MATERIALS AND METHODS

### Cell culture

HEK293T (a human embryonic kidney cell line), HepG2, and MHCC-97L (human hepatocellular carcinoma cells) were cultured in Dulbecco's modified Eagle's medium (DMEM) supplemented with 10% fetal bovine serum (FBS), 100 U/mL penicillin, and 50 mg/mL streptomycin at 37°C in a humidified 5% CO_2_ incubator.

### CD133 purification and western blotting

HEK293T/CD133-FLAG cells were lysed at 4°C for 2 h using lysis buffer [50 mM Tris (pH 7.5),150 mM NaCl, 1% TritonX-100, 2 mM EDTA, 60 mM β-glycerophosphate, 1mM sodium orthovanadate, 20 mM NaF, 10 mg/mL aprotinin, 10 mg/mL leupeptin, and 1 mM Phenylmethanesulfonyl fluoride (PMSF)]. After lysis, the insoluble material was removed by centrifugation at 12,000 *g* for 15 min. The lysates were precleared by incubation with protein G-Sepharose beads (Sigma) at 4°C for 2h. Anti-FLAG antibody-conjugated agarose gel (M2, Sigma) was incubated with the cell lysates overnight under constant agitation at 4°C. After incubation, the beads with the anti-FLAG antibody were washed three times in lysis buffer to eliminate nonspecific binding. Then, the beads were boiled for 10 min, and two bead volumes of 1% sodium dodecyl sulfate (SDS) in 20 mM phosphate-buffered saline (PBS) were added. The immunoprecipitates were resolved by SDS-polyacrylamide gels (SDS-PAGE) under reducing conditions using 8% gels. After electrophoresis, the resolved proteins were transferred to a polyvinylidene difluoride (PVDF) membrane and probed with the appropriate antibody followed by the HRP-conjugated secondary antibody.

### Identification of N-glycosylation sites by LC-MS/MS

CD133 samples were fractionated by 8% SDS-PAGE and the protein bands were visualized by Coomassie blue staining. The 95-130 kDa lanes were excised for in-gel deglycosylation. CD133 gel pieces were soaked in 50 mM ammonium bicarbonate containing 100 U/mL PNGase F overnight at 37°C. Then, the gel pieces were dried with 100% ACN and rehydrated with 10 ng/μL trypsin (sequencing grade, Promega) in 25 mM ammonium bicarbonate or 25 ng/μL chymotrypsin (Roche Molecular Biochemicals). Enzymatic digestion was performed overnight at 37°C (trypsin) or 25°C (chymotrypsin). The deglycosylated peptides were analyzed by a LC-20AB system (Shimadzu) connected to an LTQ-Orbitrap XL mass spectrometer (ThermoFisher) interfaced with an online nano-electrospray ion source (MichromBioresources). All MS/MS datas were searched against the human SWISS-PROT database using the Sequest algorithm incorporated into Bioworks software (Version 3.3.1).

### Mutation of N-glycosylation sites by site-directed mutagenesis

The nine CD133 N-glycosylation sites were mutated to Gln by sequence overlap extension PCR. All mutations were verified by DNA sequencing to ensure the presence of the correct mutation and the absence of any other randomly introduced mutations.

### Lentivirus production and hepatoma cell infection

For expression of human CD133 in hepatoma cells, the LV-CD133-FLAG plasmid was constructed by inserting full-length human CD133 cDNA into the LV-FLAG lentiviral vector between the BamHI and AgeI sites. The lentiviral vector was co-transfected with the packaging vector into HEK293T cells by the calcium phosphate co-precipitation method to produce viruses. At 2 days after transfection, viral supernatants were collected, filtered, and concentrated by ultracentrifugation. Hepatoma cells were infected with the lentivirus in the presence of 8 μg/mL polybrene and then analyzed by western blotting.

### Flow cytometry

Cell surface CD133 expression was analyzed by flow cytometry using the phycoerythrin (PE)-conjugated CD133/1 clone AC133 antibody (Miltenyi Biotec) according to the manufacturer's protocol and a FACSAria (Becton Dickinson). A PE-conjugated isotype control monoclonal antibody (Abcam) served as the control.

### Cell proliferation assay

Cells were seeded into 96-well plates in culture medium containing 10% FBS. Cell viability was determined by an MTT assay (wavelength: 450 nm) at various time points.

### Colony formation assay

Cells infected with lentiviruses carrying control, wild-type CD133, or the N548Q mutant were seeded in six-well plates at 1×10^3^ per well. After 2 weeks, the cells were washed twice with PBS, fixed with methanol/acetic acid (3:1, v/v), and stained with 0.5% crystal violet. The number of colonies was counted under a microscope.

### Statistical analysis

For analysis of experimental data, comparison of categorical data was carried out by Student's *t*-test. Data are presented as the mean ± S.D. All *t*-tests were two-sided, and the values were considered significant for *p*-value < 0.05 in all experiments.

## SUPPLEMENTARY MATERIAL


