# Supplementary material for: Mutation of N-linked glycosylation at Asn548 in CD133 decreases its ability to promote hepatoma cell growth
Source: Oncotarget. 2015 May 12;6(24):20650–60. doi: 10.18632/oncotarget.4115 (PMC4653032; doi:10.18632/oncotarget.4115)
Supplement: Supplementary file 1 [file oncotarget-06-20650-s001.pdf]

Mutation of N-linked glycosylation at Asn548 in CD133 decreases its ability to promote hepatoma cell growth

Supplementary Material

MATERIALS AND METHODS

Reagents and antibodies

A subcellular structure localization kit (SC100) was purchased from CHEMICON. Lipofectamine 2000 (11668-027) was obtained from Invitrogen. Mouse anti-β-actin antibody (A 5441) was from Sigma-Aldrich. Goat anti-mouse-HRP secondary antibody (1858413) was from PIERCE. Human monoclonal anti-CD133/1 (AC133) antibody (130-080-801) was from Miltenyi Biotec. Mouse anti-β-Catenin antibody (610153) and mouse monoclonal anti-CyclinD1 antibody (556470) were from BD Biosciences. Rabbit polyclonal anti-FLAG antibody (F7425) was from Sigma.

In-gel tryptic digestion of CD133

The band of interest was excised from the gel, rinsed three times with Milli-Q water and then cut into approximately 1 mm^2 pieces and dried. The gel slices were reduced and alkylated with 10 mM Dithiothreitol (DTT) (Sigma) and 100 mM iodoacetamide (Sigma), respectively. Digestion was performed in 30 μL 50 mM NH_4HCO_3 solution containing 0.16 μg of sequencing grade modified trypsin, incubated for 12 h at 37 °C. The digestion products were extracted sequentially by adding 100 μL 5% aqueous trifluoroacetic acid (TFA), 0.1% TFA in 50% Acetonitrile (ACN) and 2% TFA in 50% ACN. All the extracts were combined and lyophilized for later LC and MS analysis.

Structure identification of glycopeptides by MS/MS

MS analysis were performed on an MALDI QIT TOF MS (Shimadzu), equipped with a 337 nm 166 nitrogen laser in positive ion detection. 2,5-dihydroxybenzoic acid (DHB) (12.5 mg mL^{-1}) in acetonitrile/0.1% trifluoroacetic acid (1:1) was used as the matrix; MS/MS fragmentation was achieved by CID using argon as the collision gas. For MALDI mass spectra (2 shots/profile), approximately 100 laser shots were averaged to generate an MS spectrum and 200 laser shots were averaged to generate a MALDI-MS/MS spectrum. TOFMix™ (LaserBio Labs, France) was used for external calibration of the MS. In MS data on CD133 digestion was searched against the NCBInr protein database by means of the Mascot peptide mass fingerprint search engine, using an MS tolerance of 0.05 Da. Database searched is SWISS-PROT.

Immunoprecipitation assay

Cells were lysed with RIPA lysis buffer containing 50 mM Tris (pH = 7.5), 150 mM NaCl, 1% TritonX-100, 2 mM EDTA, 60 mM β-glycerophosphate, 1mM sodium orthovanadate, 20 mM NaF, 10 mg/mL aprotinin, 10 mg/mL leupeptin and 1 mM PMSF. For immunoprecipitation analysis, the cell lysates were pre-cleared with protein G agarose beads (Sigma) for 2 h at 4 °C. Then, the anti-FLAG antibody-conjugated agarose gel (M2, Sigma) was incubated with the cell lysates overnight at 4 °C. After incubation, the beads with the anti-FLAG antibodies were washed three times with the lysis buffer. Then, beads were boiled for 10 min and two beads volumes of 1% SDS in 20 mM phosphate-buffered saline (PBS) were added. The products were subjected to SDS-PAGE and were analyzed by Coomassie Blue staining or western blotting using the indicated antibody.

Western analysis

Cell extracts were prepared with cell lysis buffer (10 mM Tris-HCl, pH 7.4, 1% SDS, 1 mM Na_3VO_4 and phosphatase inhibitor cocktail). Protein samples from the equal amounts of cell extracts were separated on SDS-polyacrylamide gels (SDS-PAGE). After electrophoresis, proteins were transferred to pretreated polyvinylidene difluoride (PVDF) membranes (Roche). The membranes were incubated for 1 h at room temperature in blocking buffer (5% non-fat dry milk in TBS containing 0.1% Tween) and subsequently for 12 h at 4 °C in blocking buffer with primary antibodies. Primary antibodies included: rabbit polyclonal anti-Src (Cell Signaling; 1:1,000), mouse monoclonal anti-CD133 (W6B3C1 clone) (Miltenyi Biotec; 1:1,000), rabbit polyclonal anti-FLAG (Sigma; 1:3,000). After several washes, the membranes were incubate for 1 h at room temperature with HRP-conjugated goat anti-rabbit antibody (Santa Cruz Biotechnology; 1:3,000) or goat anti-mouse antibody (Santa Cruz Biotechnology; 1:2,000), and signal was detected by enhanced chemiluminescence substrate (Pierce Biotechnology). For quantification, the western blot films were scanned and were densitometrically analyzed using Image J Version 1.33u software.

Cell proliferation assay

Cell proliferation was measured using the MTT assay. Cells were suspended and diluted to 1×10^4 cells/mL in fresh medium, and seeded on 96-well plates (100 µL/well). The plates with cells were cultured for a total of 3 days. For measurement, 20 µL of 3-(4,5-Dimethylthiazol-2-yl)-2,5-Diphenyltetrazolium Bromide (MTT) (5 mg/mL) was added to each well, and the cells were incubated at 37 °C for another 4 h. Then the culture medium was replaced with 150 µL dimethyl sulfoxide (DMSO, Amresco, USA). After incubating the plates on a shaker for 10 min, the absorbance was detected at 450 nm using a multilabel plate reader (Bio-Rad 3350).

Immunofluorescence

HEK293T cells transiently expressing wild-type or mutant CD133 were grown on cover glasses, fixed using freshly prepared 4% paraformaldehyde (PFA) for 20 min at room temperature, washed three times with PBS, and then blocked with a PBS-based solution containing 5% normal serum and 0.3% Triton X-100. Cells were incubated overnight at 4 °C with mouse monoclonal anti-CD133 (W6B3C1 clone) (Miltenyi Biotec; 1:1,000). After being washed three times with PBS, cells were incubated with ER stain (CHEMICON; 1:3,000) for 30-45 minutes at 37 °C in a humidified chamber. Cells were washed three times with PBS and incubated with the appropriate secondary antibody: goat anti-rabbit Alexa594 IgG (Invitrogen; 1:400). Nuclei were counterstained with Hoechst 33258 (Sigma;10μg/mL). Immunouorescent images were collected on a Leica TCS SP5 confocal microscope and analyzed using LAS AF software.

Flow cytometry assay

Cells (1×10^5/mL) were cultured in each well of 6-well plates till 70-80% confluence with normal culture medium. The cells were harvested and fixed with paraformaldehyde for 1 h and washed twice with PBS. Subsequently, the cells stained with phycoerythrin (PE)-conjugated CD133/1 clone AC133 antibody (Miltenyi Biotec, Gladbach, Germany) at a dilution of 1:100 for 30 min on ice in the dark. After washing twice with PBS, the expression of CD133 was analyzed using a FACSAria (Becton Dickinson). The results are representative of three independent experiments.

Transient transfection

Cells were cultured in 24-well plates in DMEM with 5% FBS to 80% confluence. LV-FLAG, wild-type CD133 or its N-glycosylation site mutant plasmid DNA and 2 μL Lipofectamine 2000 (Invitrogen) were diluted in 50 μL OPTI-MEM medium. Mixed gently and incubated for 5 min at room temperature. The diluted DNA and Lipofectamine 2000 solutions were mixed at a 1:1 ratio and then incubated for 20 min at room temperature. DNA-reagent complexes were added to each culture well. After 48 h, the cells were rinsed with PBS, lysed with 100 μL lysis buffer and analyzed by western analysis.

TOP/FOP luciferase reporter assay

To assess the effect of CD133 on the transcriptional activity of β-catenin, we employed the TOP/FOP reporter system using the dual-luciferase kit (Dual-Glo™Luciferase Assay System, Promega, Madison, WI, USA). Cells were transiently transfected with 1 ng of pRL-SV40 vector encoding *Renilla* luciferase (Promega) and 200 ng of β-catenin-responsive

firefly luciferase reporter plasmid Top*Flash* or the negative control Fop*Flas*. After 72 h, cells were lysed with 1× reporter lysis buffer (Promega). Both firefly and *Renilla* luciferase activity was measured according to the manufacturer's instructions. The firefly luciferase activity was normalized against the *Renilla* luciferase activity.
